# The Oral Bioavailability of Vitamin B_12_ at Different Doses in Healthy Indian Adults

**DOI:** 10.3390/nu16234157

**Published:** 2024-11-30

**Authors:** Sindhu Kashyap, Poorvikha Gowda, Roshini M. Pasanna, Ambily Sivadas, Harshpal S. Sachdev, Anura V. Kurpad, Sarita Devi

**Affiliations:** 1 Division of Nutrition, St. John’s Research Institute, St. John’s National Academy of Health Sciences, Bangalore 560034, India; sindhu.k@sjri.res.in (S.K.); roshni.mp@sjri.res.in (R.M.P.); 2 St. John’s Medical College, St. John’s National Academy of Health Sciences, Bangalore 560034, India; poorvikha.s@stjohns.in; 3 Department of Molecular Biology and Genetics, St. John’s Medical College Hospital, St. John’s National Academy of Health Sciences, Bangalore 560034, India; ambily.s@sjri.res.in; 4 Department of Paediatrics, Sitaram Bhartia Institute of Science and Research, New Delhi 110016, India; hpssachdev@gmail.com; 5 Department of Physiology, St. John’s Medical College, St. John’s National Academy of Health Sciences, Bangalore 560034, India; a.kurpad@sjri.res.in

**Keywords:** vitamin B_12_, bioavailability, [^13^C]-cyanocobalamin, dose dependency

## Abstract

Background/Objectives: The bioavailability of crystalline vitamin B_12_ (B_12_) through active absorption is reported to have a maximum capacity of 1.5–2.5 µg per dose. A small passive bioavailability has also been suggested at high doses. The present study aimed to determine the dose-dependency of active B_12_ absorption and to quantify its passive absorption at higher doses. Methods: The dose-dependency of crystalline B_12_ bioavailability was determined in nine healthy adults, using oral [^13^C]-cyanocobalamin, in a cross-over design at doses of 2.5, 5, and 10 µg. The dose order was randomised, with a washout of one month. Literature data from was added to the present study data in a meta-analysis of the relation between B_12_ bioavailability and dose, to evaluate its pattern at different doses. Results: Bioavailability, as a function of dose, was significantly different between 2.5, 5, and 10 µg doses of [^13^C]-cyanocobalamin at 50.9 ± 32.5%, 26.7 ± 22.3%, 15.4 ± 13.6%, respectively, (*p* < 0.01), while the absolute bioavailability trended upward, at 1.16 ± 0.74 µg, 1.22 ± 1.02 µg, and 1.39 ± 1.23 µg (*p* = 0.46). The meta-analysis showed two distinct phases of bioavailability. Up to a dose of 2.6 µg, there was a significant steep positive correlation, with a slope (bioavailability) of 43%/µg suggesting an active process with a maximum of 1.2 µg. At higher doses, the slope was 1%/µg, not significantly different from zero, possibly a passive process. Conclusions: The active bioavailability of crystalline B_12_ is not dose-dependent, saturating at ~1.2 µg.

## 1. Introduction

Vitamin B_12_ (B_12_, cobalamin) is a co-factor for two important enzymes, methionine synthase and methylmalonyl CoA mutase, which are involved in conversion of homocysteine to methionine and methylmalonyl CoA to succinyl CoA, respectively [1,2]. The former reaction leading to the formation of methionine is required for production of S-adenosyl methionine, which is an essential methyl donor for several methyl transferases and is required in deoxyribonucleic acid (DNA) synthesis, erythropoiesis, and myelinogenesis [3,4]. Deficiency of B_12_ not only leads to depletion of methyl groups for methylation of DNA, histones, and gene expression regulators but also to the accumulation of homocysteine and methylmalonic acid, which could lead to cellular stress and apoptosis [5,6]. Hyper homocysteinemia is also shown to be proatherogenic [7]. The clinical manifestation of B_12_ deficiency can be hematological leading to macrocytic and megaloblastic anemia, while neurological symptoms include sensory loss, motor disturbances, and cognitive impairment, and severe deficiency can be characterised by psychosis, paranoia, and severe depression [8,9]. Neurological manifestations of B_12_ deficiency are mainly related to demyelination of neurons, which can occur early and in isolation of hematological complications [9]. In several life stages where the requirement is high such as pregnancy and lactation, infancy, childhood and adolescence, and in later years, the risk of deficiency is higher [10]. Deficiency of B_12_ during pregnancy could lead to a neural tube defect in the infant [10].

There are several causes of B_12_ deficiency that could be linked to any one of the steps involved in its absorption, inherited defects in transport of B_12_, or due to inadequate dietary intake [11]. The absorption of vitamin B_12_ (B_12_, cobalamin) is complex and involves multiple processes starting from its dissociation from the food protein in the stomach by the action of pepsin and hydrochloric acid [12]. Free B_12_ then binds to haptocorrin (R-protein) secreted by salivary glands and gastric mucosa. Haptocorrin is degraded by pancreatic proteases in the duodenum and freed B_12_ complexes with intrinsic factor (IF), a protein secreted by the parietal cells [13]. The IF–B_12_ complex on reaching the distal ileum binds to the cubulin receptor on the ileal mucosa, and this complex is internalised through receptor-mediated endocytosis [14]. This process is saturable [15], as it is mainly dependent on the capacity of the ileal cubulin receptors [16]. However, there are reports of resolution of functional symptoms of deficiency at massive doses of 500 mg/day in the elderly, or 1000 µg/day in pernicious anemia, which could probably be attributed to a small but significant passive absorption [17,18]. This is thought to be in the order of 1%, but there has been no quantitative measurement of this process.

The estimated average requirement (EAR) of B_12_ in adults aged 19 to 70 y is 2.0 µg/day [19]. While this can be met in most diets, in populations with lower dietary B_12_ intake, or in populations that may have higher requirements due to lower absorption capacity, such as atrophic gastritis in older adults, supplementation or fortification of foods is recommended [11]. Understanding the limit of absorption of crystalline B_12_ therefore becomes important. There are not many recent studies that have examined the absorption of vitamin B_12_ in response to different doses. One study that administered crystalline radioactive ^58^Co-cyanocobalamin at 1 µg, 5 µg, and 25 µg showed absolute absorptions of 0.5 µg, 1 µg, and 1.4 µg, respectively [20]. This would mean that the absorption is both low and somewhat dose-dependent. Similarly, our earlier stable isotope labelled [^13^C]-cyanocobalamin bioavailability study also showed that the bioavailability from a 2.3 µg dose was 1.06 µg and from an 18.3 µg dose was 1.39 µg, or 46.2% and 7.6%, respectively [21,22]. These observations also fit in with the observations from several previous studies, which indicated the maximum threshold of B_12_ absorption to be between 1.5–2.5 µg per dose ingested [23]. However, these dose studies were conducted in different participants with different B_12_ statuses, and therefore conclusions drawn on the proportion absorbed may not be directly extrapolatable.

In addition, the use of radiolabelled B_12_ to measure absorption has its own inherent limitations including the atom labelled, which is cobalt (Co) in most cases and therefore the radioactivity measured can be of non-active B_12_ analogs. An alternative for this is the use of ^14^C labelling of the B_12_ carbon skeleton; however, there is still a minimal radiation risk to the participant [24]. A relatively novel method, which offers a safe non-radioactive substitute for measuring B_12_ bioavailability, uses a ^13^C-stable isotopically labelled cyanocobalamin synthesised from *Salmonella enterica* [21]. The present study therefore aimed to assess dose dependency of B_12_ absorption using [^13^C]-cyanocobalamin in the same participants in a paired manner, to establish the absolute amount absorbed from a single ingested dose and to quantify what the passive absorption could be with higher doses.

## 2. Methods

**Figure 1:**
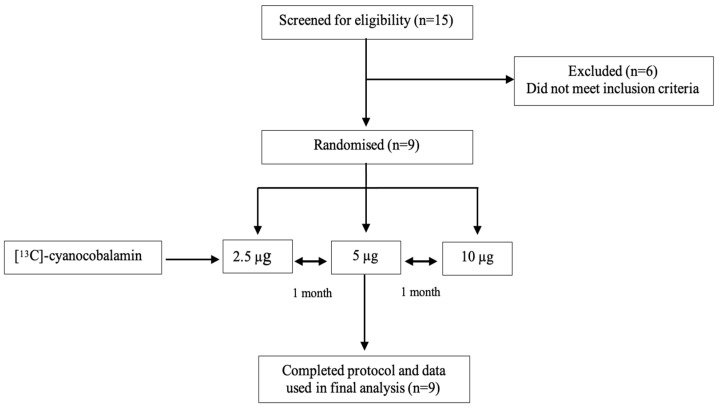
Consort flow chart with participant screening and study protocol.

A cross-over study was conducted in humans to assess the oral dose dependency of B_12_ bioavailability. Bioavailability studies for 2.5 µg, 5 µg, and 10 µg [^13^C]-cyanocobalamin were conducted on three randomly assigned experiment days separated by one month for each participant. Healthy Indian participants (*n* = 9) aged between 18 and 40 y, with BMIs of 18.5 to 25 kg/m^2^, no history of major comorbid illnesses including gastrointestinal disease, and not on any chronic medication and vitamin supplementation within 4 weeks of the study were included in the study. Participants who were anaemic, had B_12_ deficiency, had a history of long-term (>4 weeks) use of NSAIDs, used proton pump inhibitors and H_2_ receptor blockers, had recent antibiotic use, or binged alcohol were excluded. The drug history was re-checked prior to each experiment day, and the participants were instructed not to take any vitamin supplementation throughout the course of the study. The Institutional Ethical Review Board of St. John’s Medical College, Bengaluru, India, approved the study, and written informed consent was obtained from all the participants at enrolment. The participant recruitment details and study protocol are represented in Figure 1.

The [^13^C]-cyanocobalamin tracer used was synthesised as a batch process for an earlier study, and the details of synthesis, preparation and characterisation of the tracer has been detailed elsewhere [21]. Briefly, a seed culture of *Salmonella enterica* was grown in no-carbon essential medium supplemented with glutamic acid (Sigma Aldrich, Darmstadt, Germany) and ferric chloride (HIMEDIA, Mumbai, India) and incubated for 12 h at 37 °C. Production flasks containing culture medium with the labelled precursor ethanol-[^13^C_2_]-amine (40 mM, Sercon Ltd., Cheshire, UK), dicyanocobinamide (250 nM, Sigma Aldrich, Darmstadt, Germany), and 500 nM of dimethylbenzimidazole (DMB, Sigma Aldrich, Darmstadt, Germany) were inoculated with bacteria in the seed culture and incubated in the dark at 37 °C for 48 h. Cells were harvested by centrifugation (5810 R, Eppendorf, Enfield, CT, USA), resuspended in methanol (Honeywell, Offenbach am Main, Germany) and sodium cyanide (Acros Organics, Belgium, WI, USA), and incubated in a shaking water bath at 65 °C overnight. The solution was centrifuged (5810 R, Eppendorf, Enfield, CT, USA) and filtered (Sartorius, Gottingen, Germany), the filtrate was evaporated to dryness (Labconco, Kansas City, MO, USA), and the residue was dissolved in sterile distilled water [21]. This was purified again by high-performance liquid chromatography (HPLC, UV detector, Shimadzu, Kyoto, Japan), and purified extracts were stored (−80 °C). During characterisation of the molecule with high-resolution accurate mass spectrometry analysis (Q Exactive, LC-HRAM-MS; Thermo Scientific, Waltham, MA, USA), the most intense peak observed was a 4-Dalton shift for [^13^C]-cyanocobalamin compared with standard cyanocobalamin, which was chosen for the bioavailability estimation [21].

The participants reported to the metabolic ward of St. John’s Medical College Hospital after an overnight fast of 10 h. An intravenous catheter (Jelco 22 G; Medex Medical Ltd., Kent, UK) was inserted into their antecubital vein for blood sampling, and a baseline blood sample was collected. A bolus dose of oral tracer [^13^C]-cyanocobalamin (2.5 µg, 5 µg or 10 µg) was administered in randomly generated order, and blood samples were collected at 1, 5, 6, and 7 h post tracer dosing.

**Figure 2:**
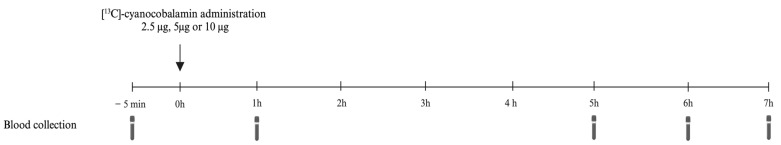
Experiment day protocol followed for each dose day in the oral dose dependency of the B_12_ bioavailability study.

The participants were not allowed to consume any food or drink except water during the entire study duration. The time of blood collection was decided based on the peak plasma isotopic concentrations of [^13^C]-methylcobalamin in a previous oral [^13^C]-cyanocobalamin kinetics study [21]. This reduced the sampling burden on the participants of the current study. The study day protocol is represented in Figure 2.

Whole blood samples were collected in aluminum foil-wrapped EDTA vacutainers (Becton Dickinson, Franklin Lakes, NJ, USA), processed for plasma separation immediately by centrifugation (5810 R; Eppendorf, Enfield, CT, USA) at 2588× *g* for 10 min at 4 °C, and stored at −80 °C until analysis. The analysis of different forms of [^13^C]-cobalamin (cyano, methyl, ado and hydroxo) was performed as described earlier [21]. Briefly, plasma samples (1 mL) were spiked with 10 μL internal standard (IS; methotrexate, 0.20 μmol/L; Sigma-Aldrich, Burlington, MA, USA), vortex mixed for 10 s, and acidified by the addition of 175 μL formic acid (Sigma-Aldrich, Burlington, MA, USA) before deproteinization. A 4-fold of chilled organic solvent (100% acetonitrile, liquid chromatography mass spectrometry (LCMS grade; Honeywell, Pottsville, PA, USA) was added to each sample, vortexed vigorously, and kept at 4 °C for 10 min, followed by centrifugation (5810 R; Eppendorf, Enfield, CT, USA) at 15,294× *g* for 30 min at 4 °C. Acidified supernatants were dried in a vacuum concentrator (Labconco, Kansas City, MO, USA) at 30 °C for 7 h. All sample preparation was performed in the dark. Dried samples were reconstituted in 200 μL water (LCMS grade; Honeywell, Pottsville, PA, USA), and analysis was performed on a high-resolution analytical platform consisting of a Vanquish Flex Binary UHPLC coupled to a mass spectrometer (Q Exactive, LC-HRAM-MS; Thermo Scientific, Waltham, MA, USA) with a heated electrospray ionization (HESI-II) probe. Various forms of vitamin B_12_ were separated on a Hypersil Gold aQ column (100 × 2.1 × 1.9 μm; Thermo Scientific, Waltham, MA, USA) [21]. A reversed-phase-gradient elution at 0.3 mL/min was used to deliver the mobile phase, which consisted of water with 0.1% formic acid (eluent A) and acetonitrile with 0.1% formic acid (eluent B). The gradient used was 5% B at 0–2 min, which was increased to 50% B at 22 min, then increased to 98% B at 23 min, held for 2 min, then decreased to 5% B at 26 min, and equilibrated for another 7 min [21]. The column temperature was maintained at 40 °C, and the injection volume was 50 μL for solvent blanks, standards, and samples [21].

**Figure 3:**
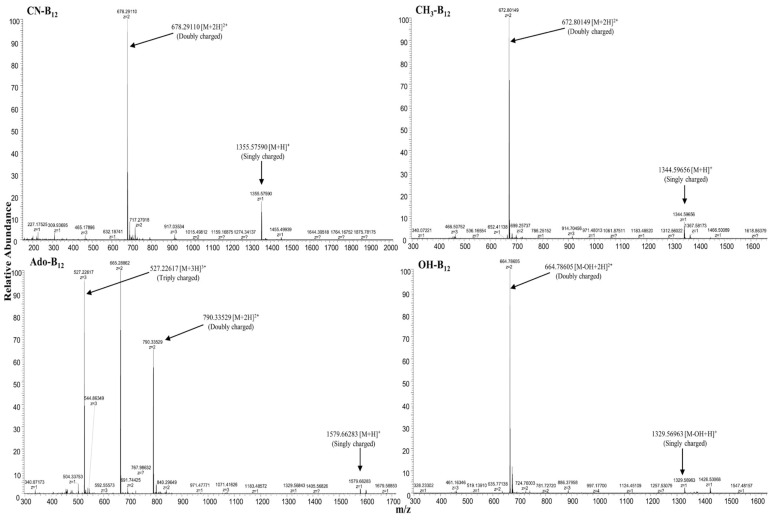
High-resolution accurate mass spectrometry analysis of CN (cyano)-, Ado (adenosyl)-, CH3 (methyl)-, and OH (hydroxo)-vitamin B-12 (singly, doubly, and triply charged species). The precursor masses at *m*/*z* 678.29098 and *m*/*z* 680.29738, *m*/*z* 672.80149 and *m*/*z* 674.80149, *m*/*z* 664.78568 and *m*/*z* 666.78568, and *m*/*z* 790.33645 and *m*/*z* 792.33645 for [^12^C]- and [^13^C]-cobalamin species of cyano-, methyl-, hydroxo-, and adenosylcobalamin, respectively. Figure reproduced from Ref. [21].

**Figure 4:**
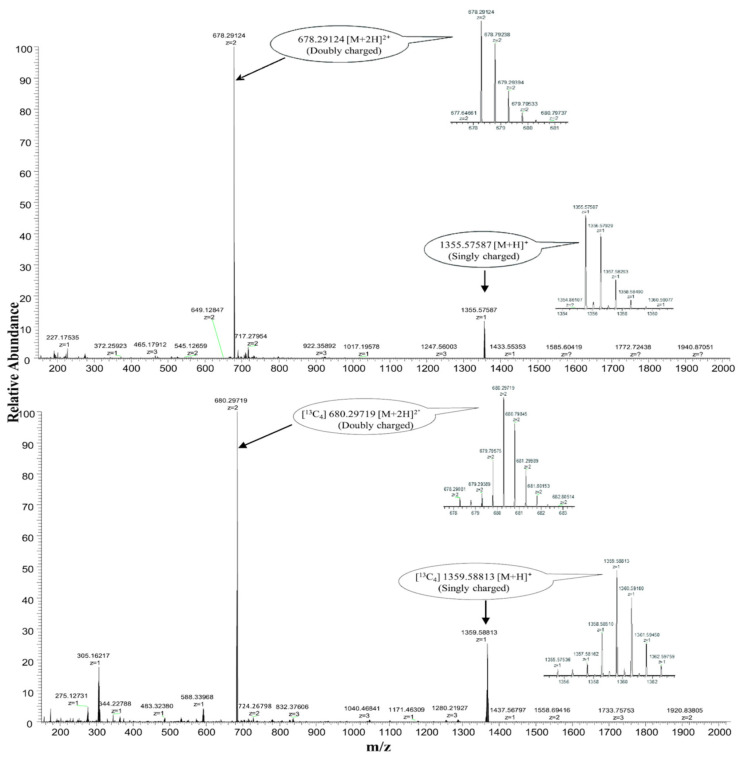
High-resolution accurate mass spectrometry analysis of the synthesised [^13^C]-cyanocobalamin showing doubly charged ion at *m*/z 680.29768 [M + 2H]^2⁺^ with the 4-Dalton shift as compared with standard cyanocobalamin. Figure reproduced from Ref. [21].

Mass spectrometer source parameters were as follows: sheath gas flow rate: 40; auxiliary gas flow rate: 10; spray voltage: 4.0 kV; capillary temperature: 330 °C; heater temperature: 350 °C; and S-Lens RF level 60. The data were acquired by using Thermo Scientific Xcalibur software (Version 4.1.31.9) [21]. The quantification was performed for double-charged species of cyano-, methyl-, hydroxo-, and adenosylcobalamin (Figure 3 and Figure 4). The precursor (product ions) masses monitored were 678.29098 (147.09164, 912.44135) and 680.29738 (147.09164, 914.44135), 672.80149 (147.09164, 971.47383) and 674.80149 (147.09164, 973.47383), 664.78568 (147.09174, 912.44165) and 666.78568 (147.09174, 914.44165), and 790.33645 (147.09165, 971.47968) and 792.33645 (147.09164, 973.47968) for [^12^C]- and [^13^C]-cobalamin species for cyano-, methyl-, hydroxo- and adenosylcobalamin, respectively, and 455.17859 (134.06004, 175.07263, 308.12527) for methotrexate. Standards in the range of 50–2000 pmol/L were used for quantification and were linear in this range (r^2^ = 0.9979), and the reproducibility was <8% [21]. The ratios of peak area of respective cobalamins and IS were plotted against their concentrations. The concentrations of the samples were calculated from the regression equations of individual cobalamin forms. The intra- and inter-assay CVs were <6% and <8% [21]. Only [^13^C]-methylcobalamin tracer was detected, and these data were plotted against time.

B_12_ bioavailability was estimated by modelling the [^13^C]-methylcobalamin time-concentration profiles using a two-compartment model with a double zero-order absorption process using the nonlinear mixed-effect modelling software, Monolix 2019R2, (Lixoft, Antony, France) as described earlier [21]. The mathematical model was based on the following equations:

$$\frac{dC_{1}}{dt}={Ab}_{1}\left(t\right)+{Ab}_{2}\left(t\right)-kC_{1}-k_{12}C_{1}+k_{21}C_{2}$$

$$\frac{dC_{2}}{dt}={k_{12}C_{1}-k}_{21}C_{2}$$

The absorption functions followed the general form:

$$Ab_{1}\left(t\right)=\frac{\;f\%\cdot D\cdot F1}{V\cdot Tk_{01}},\;Tk_{01}\geq t\;\geq 0$$

$$Ab_{2}\left(t\right)=\left\{\begin{array}{c} \;0,\;t<Tlag2\\ \;\frac{f\%\cdot D\cdot (1-F1)}{V\cdot Tk_{02}},\;Tlag2+Tk_{02}\geq t\;\geq Tlag2 \end{array}\right.$$

where *C*_1_ and *C*_2_ represent the tracer concentrations in the plasma pool and the tissue storage pool, respectively; *Ab*_1_ and *Ab*_2_ are the absorbed tracer concentrations from the first (1st hour post dose) and second absorption phases (5th to 7th hours post dose), respectively; *k* is the elimination coefficient; *k*_12_ and *k*_21_ are the transfer constants between the plasma and the tissue storage pools; *f*_%_ is the fractional bioavailability; *D* is the total tracer dose administered; *F*_1_ is the first-phase absorption fraction; *V* is the volume of distribution; *Tk*_01_ and *Tk*_02_ are the durations of first and second absorption phases, respectively; and *Tlag*_2_ is the delay before the second absorption phase [21].

Mathematically, the terms $\frac{f\%}{V}$ are irreducible. To circumvent this, we calculated an apparent volume of distribution (*V_app_ =* $\frac{V}{f\%}$) using the dose administered [21]. The fractional bioavailability was rescaled using plasma volume (*V_p_*) as the volume of distribution. Fractional bioavailability (f%*)* could be mathematically arranged as:

f%=DoseVpDoseVapp

which reduces to:

$$f\%=\frac{V_{p}}{V_{app}}$$

where *V_p_* was taken as 40 mL/kg body weight [21].

To avoid overfitting, Bayesian estimation was used to set the initial parameter values using prior information from our previous study.

### Statistical Analyses

The sample size of 9 healthy adults was sufficient to evaluate a significant change of fractional bioavailability by 25% between doses with a SD of 15% at *p* < 0.01 and power of 80%. This sample size also allowed for multiple comparisons between 3 doses.

Data are presented as mean ± SD to allow comparisons with previous studies. Since the mean and median of both fractional and absolute bioavailability for each B_12_ dose were not similar, and the SD was more than half mean, non-parametric tests were used for data analyses. Comparison of B_12_ fractional and absolute bioavailability between the three oral doses was performed using the Freidman’s test of repeated measures with a post-hoc Durbin–Conover test.

To assess whether absolute bioavailability of cyanocobalamin remained linear or reached a threshold and plateaued with the administered dose, we compiled absolute bioavailability from previous studies (that used radiolabelled cyanocobalamin) with oral doses up to 50 µg [20,21,25,26], and we combined these with the data from the present study (Table 1). When the data were examined visually, the absolute bioavailability showed two distinct phases when plotted against the B_12_ dose, with a much higher positive slope at lower doses and a much lower slope at higher doses. To determine the break-point between these two phases, a piecewise linear regression model was performed for the relation between absolute bioavailability and B_12_ dose [27]. The statistical significance of the break-point was assessed using Davies test [28]. The delta method was used to determine the standard error and 95% CI of the break-point estimate [29].

## 3. Results

**Figure 5:**
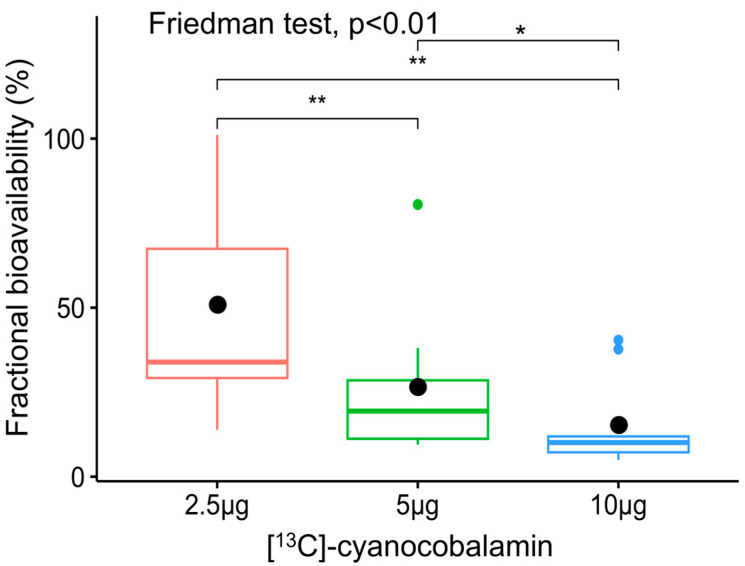
Fractional bioavailability of [^13^C]-cyanocobalamin at oral doses of 2.5 µg, 5 µg, and 10 µg (*n* = 9). The vertical lines that spilt each box represent the median, the whiskers represent the interquartile ranges, and the filled black circles indicate mean fractional bioavailability of each oral dose. ** *p* < 0.01 and * *p* < 0.05.

**Figure 6:**
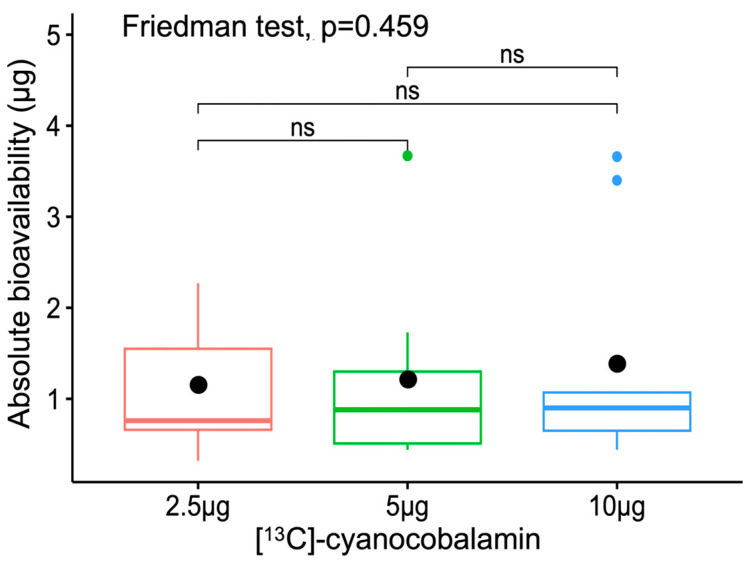
Absolute bioavailability of [^13^C]-cyanocobalamin at oral doses of 2.5 µg, 5 µg, and 10 µg (*n* = 9). The vertical lines that spilt each box represent the median, the whiskers represent the interquartile ranges, and the filled black circles indicate mean absolute bioavailability of each oral dose. ns: not significant, *p* > 0.05.

The participant details of body mass index (BMI), anthropometry, haemoglobin, and serum active B_12_ are represented in Table 2. All the participants had normal BMI (18.5 to 25.0 kg/m^2^), were not anaemic, and were B_12_ sufficient (25.1 to 165 pmol/L) as indicated by their serum active B_12_ (holotranscobalamin) concentrations. The individual participants’ [^13^C]-methylcobalamin plasma concentration time profiles at different doses and pharmacokinetic parameters are provided in Supplementary Figure S1 and Supplementary Tables S1 and S2. The fractional bioavailability decreased significantly with increasing doses of [^13^C]-cyanocobalamin, where it was 50.9 ± 32.5%, 26.7 ± 22.3%, and 15.4 ± 13.6% for doses of 2.5 µg, 5 µg, and 10 µg, respectively (*p* < 0.01) (Figure 5). The absolute bioavailabilities of 2.5 µg, 5 µg, and 10 µg [^13^C]-cyanocobalamin were 1.16 ± 0.74 µg, 1.22 ± 1.02 µg, and 1.39 ± 1.23 µg, respectively, and these values, although trending upward, were not significantly different from one another (Figure 6).

**Figure 7:**
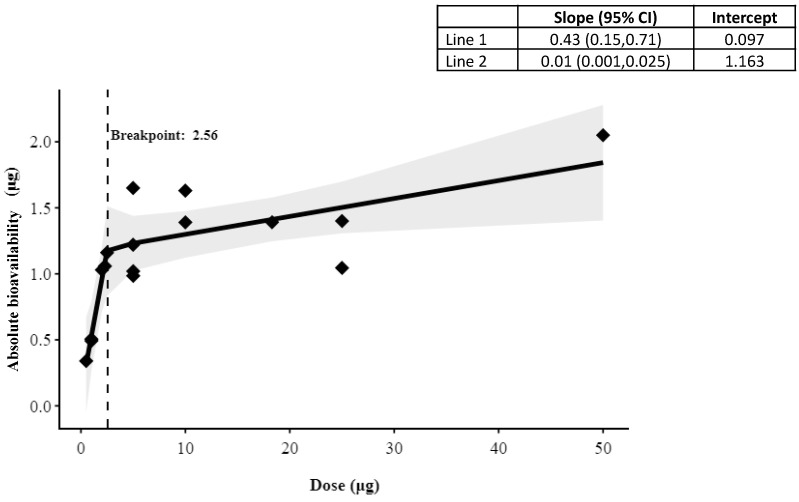
Break-point analysis of absolute bioavailability at different doses of cyanocobalamin compiled from bioavailability data of the previous and present studies.

The maximum response of absolute bioavailability in the break-point analysis was found to be at 2.56 µg oral dose, where the maximum absorption was 1.2 µg. The slopes of the relationships before and after the break-point were 0.43 (95% CI: 0.15, 0.71) and 0.01 (95% CI: 0.001, 0.025), respectively (Figure 7).

## 4. Discussion

The present study determined the bioavailability of vitamin B_12_ at different doses in the same participants. The fractional bioavailability significantly decreased with the dose (50.9 ± 32.5%, 26.7 ± 22.3%, 15.4 ± 13.6%), which is expected, as the denominator (dose) increased with the same absolute bioavailability. However, the absolute bioavailability was not significantly different among the doses (1.16 ± 0.74 µg, 1.22 ± 1.02 µg, and 1.39 ± 1.23 µg), even though there was an increasing trend. The average bioavailability across all doses was 1.25 µg.

In a previous dose-ranging study in different participants using radiolabelled 1 µg, 5 µg, and 25 µg ^58^Co-cyanocobalamin, bioavailability was measured by whole-body counting. The fractional and absolute bioavailability were 49.2 ± 14.9%, 20.4 ± 8.8%, and 5.6 ± 2.2% and 0.5 µg, 1 µg, and 1.4 µg, respectively [20]. The bioavailability of 5 µg cyanocobalamin, which was the only common dose between the studies, showed similar fractional and absolute bioavailability (20.4 ± 8.8% vs. 26.7 ± 22.3% in the present analysis, and 1.02 ± 0.44 vs. 1.22 ± 1.02 in the present analysis) [20]. The values obtained in the previous study cannot be taken as an accurate measure of bioavailability, as whole-body radioactivity counting was performed for this measurement 16 days after dosing, and the measurement of radioactivity might not necessarily be of an active cobalamin molecule [20,30]. In yet another study where 0.5 µg, 2 µg, 5 µg, and 10 µg doses of ^60^Co-B_12_ (specific form not mentioned) were administered and the amount absorbed were calculated by faecal excretion of ^60^Co, it was found that 0.34 µg, 1.03 µg, 1.65 µg, and 1.63 µg were absorbed, respectively [26]. In that study, the doses of 2 µg, 5 µg, and 10 µg were similar to those used in the present study; however, the absorbed quantity was numerically greater [26]. This higher absorption could be due to differences in absorptive capacity in different populations. Additionally, this could still be an underestimate as the daily faecal radioactivity measured to determine absorption would include losses from enterohepatic circulation [31].

The present analysis reconfirms that there is an absorption threshold of vitamin B_12_ when low doses are administered. This limit is likely due to the localization of cubulin receptors on the distal ileum and due to the delay in recycling of the endocytosed receptors back to the apical surface of the brush border of ileal enterocytes. The range of absolute bioavailability was between 0.32 µg and 2.27 µg, 0.44 µg and 3.67 µg, and 0.44 µg and 3.66 µg for 2.5 µg, 5 µg, and 10 µg oral [^13^C]-cyanocobalamin doses, respectively. The wide range of bioavailability observed here could be due to various factors including diet, undiagnosed *Helicobacter pylori* infection, and genetic factors such as polymorphisms in the CUBN gene (cubulin gene) [32,33]. For instance, in American, Icelandic, and Danish populations a variant of CUBN gene rs1801222 was associated with higher B_12_ status [34,35]. In the present study, the absolute absorption at 2.5 µg [^13^C]-cyanocobalamin dose, at which an absorption threshold was achieved, correlated positively and significantly with serum active B_12_ of the participants (Spearman’s rho = 0.678, *p* = 0.045). This re-emphasises the role of various factors mentioned above, which could act singly or in combination, influencing active B_12_ absorption and therefore the B_12_ status.

Higher oral doses of B_12_ have been shown to be effective in restoring B_12_ status and reducing functional indicators [36]. A 16-week supplementation dose-ranging study in older adults with mild B_12_ deficiency showed a 33% reduction of functional indicators such as serum methylmalonic acid (MMA) at 647 to 1032 µg cyanocobalamin administration [36]. One reason high doses work clinically in improving B_12_ status, despite the apparent limit in active absorption, is that passive absorption of B_12_ can also occur, over and above active absorption [37]. The break-point analysis performed showed a maximum absolute bioavailability (1.2 µg) at 2.56 µg dose, at which there was a small incremental increase in the absolute bioavailability with the dose. From the slope of the linear line before the break-point, the amount absorbed was 0.43 µg per 1 µg of dose till the break-point (2.56 µg dose). After the break-point, in addition to active absorption, 1% of the administered dose could be absorbed through passive absorption. When the bioavailability data of cyanocobalamin were increased from 50 µg to 1000 µg oral dose and the data were reanalysed [38], though the break-point remained similar at 2.53 µg, the bioavailability after this (passive) increased to 2.3% of the administered dose; this is equivalent to 23 µg of a 1000 µg dose (Figure 7). The evidence of effectiveness of passive absorption at higher doses was recently demonstrated in B_12_-deficient patients with pernicious anaemia, where they recovered from deficiency [18]. Although the higher doses have proven to improve B_12_ status in deficient populations, caution should be exercised in the duration of administration as the fate of the unabsorbed B_12_ and its long-term effects are currently unknown. An alternative would be to administer 2.5 µg at an interval of 4–6 h, which in the long-term would still result in improved B_12_ status as at this dose it would deliver 1 µg/intake [36].

The strengths of this study are the use of a minimally invasive, safe novel tracer, [^13^C]-cyanocobalamin, which allowed direct measurement of its bioavailability, and the cross-over design, which removed the effect of interindividual variability. A limitation is that we did not test doses beyond 10 µg; however, other earlier studies have shown that at doses of 18.3 µg or 25 µg the absorption remained similar as 2.5 µg. Another possible limitation could be the small sample size; although the confidence of the results obtained from this study can be considered to be high due to its cross-over design, this needs to be validated in a larger sample in the future. The absorption of B_12_ at different doses in the present study should be extrapolated with caution, as it was studied in one population group.

## 5. Conclusions

The present study evaluated dose dependency of crystalline B_12_ bioavailability in healthy Indian adults using oral [^13^C]-cyanocobalamin as a tracer at doses of 2.5, 5, and 10 µg in a cross-over design. The active absorption of B_12_ was found be saturable and not dose-dependent. When data from previous studies were added to the present study data, and a break-point analysis was performed on the bioavailability–dose relationship, it showed two phases, with a first phase up until a dose of 2.56 µg was reached, with a steep slope that signified an absorption of 43% of the administered dose, after which the slope was shallower, with an absorption of 1% of the administered dose probably through passive absorption. The maximum absorption, which can be considered as the active absorption threshold, was determined to be ~1.2 µg at an oral B_12_ dose of 2.5 µg.

## Figures and Tables

**Table 1:** The bioavailability of crystalline cyanocobalamin at different oral doses compiled from the literature.

Cyanocobalamin Dose (µg)	Fractional Bioavailability (%)	Absolute Bioavailability (µg)	Reference
0.5	65.0	0.34	[26]
1	50.6	0.51	[25]
1	49.2	0.49	[20]
2	52.0	1.03	[26]
2.3	46.0	1.06	[21]
5	19.7	0.99	[25]
5	20.4	1.02	[20]
5	33.0	1.65	[26]
10	16.0	1.63	[26]
18.3	7.6	1.39	[21]
25	5.6	1.40	[20]
25	4.18	1.05	[25]
50	4.1	2.05	[25]

**Table 2:** Characteristics of study participants ^1^.

Characteristics	Value
Gender (female/male)	5/4
Age (y)	20.6 ± 1.3
Height (m)	165.4 ± 8.5
Weight (kg)	60.2 ± 8.8
BMI (kg/m^2^)	21.9 ± 1.7
Hb (g/dL)	13.6 ± 1.5
Serum active vitamin B_12_ (pmol/L)	45.6 ± 25.1

^1^ Values are mean ± SD, *n* = 9.

## Data Availability

Data described in the manuscript, code book, and analytic code will be made available upon reasonable request to the corresponding author due to ethical reasons.

## Supplementary Materials

The following supporting information can be downloaded at: https://www.mdpi.com/article/10.3390/nu16234157/s1, Supplementary Figure S1. Individual fits describing the plasma [^13^C]-methylcobalamin concentration-time profiles for oral doses of 2.5 μg, 5 μg and 10 μg of [^13^C]-cyanocobalamin (*n* = 9). Randomization sequence with [^13^C]-cyanocobalamin oral dose at each visit is given in the inserted table. Table S1. Model parameter estimations for the [^13^C]-methylcobalamin plasma concentration-time profiles after oral administration of 2.5 μg, 5 μg and 10 μg of [^13^C]-cyanocobalamin (*n* = 9). Table S2. The final estimated parameters and bioavailability after oral administration of 2.5 μg, 5 μg and 10 μg of [^13^C]-cyanocobalamin (*n* = 9).

## Abbreviations

B12	Vitamin B12
BMI	Body mass index
CBC	Complete blood count
Co	Cobalt
CUBN	Cubilin
EAR	Estimated average requirement
HESI	Heated electrospray ionization
HPLC	High-performance liquid chromatography
IF	Intrinsic factor
LC-MS/MS	Liquid chromatography tandem mass spectrometry
UHPLC	Ultrahigh-performance liquid chromatography
