# Supplementary material for: The Oral Bioavailability of Vitamin B12 at Different Doses in Healthy Indian Adults
Source: Nutrients. 2024 Nov 30;16(23):4157. doi: 10.3390/nu16234157 (PMC11643782; doi:10.3390/nu16234157)
Supplement: Supplementary file 1 [file nutrients-16-04157-s001.zip › nutrients-3264350-supplementary.pdf]

The oral bioavailability of vitamin B12 at different doses in healthy Indian adults.
Sindhu Kashyap et al

Supplementary Figure S1. Individual fits describing the plasma [^{13}C]-methylcobalamin concentration-time profiles for oral doses of 2.5 µg, 5 µg and 10 µg of [^{13}C]-cyanocobalamin (n=9). Radomization sequence with [^{13}C]-cyanocobalamin oral dose at each visit is given in the inserted table.

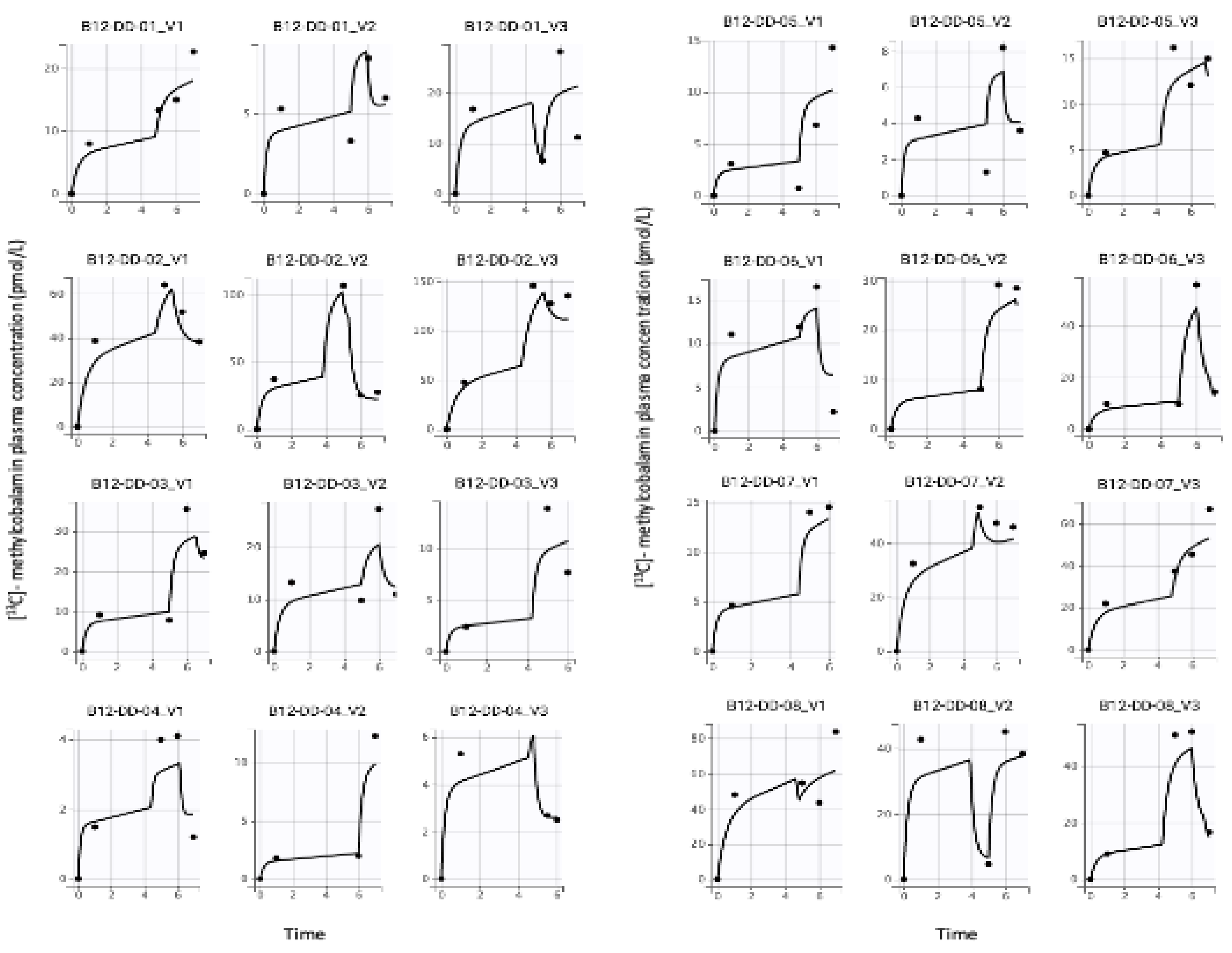

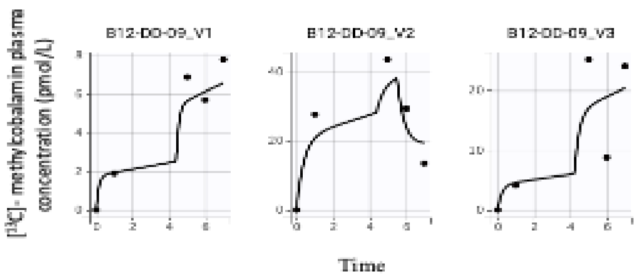

Participant ID	Randomisation sequence
B12-DD-01	V1-2.5 µg, V2-10 µg, V3-5 µg
B12-DD-02	V1-2.5 µg, V2-10 µg, V3-5 µg
B12-DD-03	V1- 10 µg, V2-2.5 µg, V3 5 µg
B12-DD-04	V1-10 µg, V2-2.5 µg, V3- 5 µg
B12-DD-05	V1-5 µg, V2-10 µg, V3-2.5 µg
B12-DD-06	V1-10 µg, V2- 5 µg, V3- 2.5µg
B12-DD-07	V1-10 µg, V2- 2.5 µg, V3-5 µg
B12-DD-08	V1- 2.5 µg, V2-10 µg, V3- 5 µg
B12-DD-09	V1-10 µg, V2-2.5 µg, V3-5 µg

*V1-Visit 1, V2-Visit 2, V3-Visit 3

Supplementary Table S1. Model parameter estimations for the [^{13}C]-methylcobalamin plasma concentration-time profiles after oral administration of 2.5 µg, 5 µg and 10 µg of [^{13}C]-cyanocobalamin (n=9).

**Parameter (units)**	**Estimate (%RSE)**	**%BSV (%RSE)**
Tk01 (h)	6.29 (33)	36.1 (61.4)
Tk02 (h)	3.1 (NC)	100 (NC)
F1	0.6 (NC)	56.9 (NC)
Tlag2 (h)	4.43 (NC)	23.6 (46.4)
V (L)	9.91 (80.9)	99.5 (45)
k (h^{-1})	0.00205 (230)	196 (95)
k12 (h^{-1})	3.14 (63.1)	79.5 (42.5)
k21 (h^{-1})	0.0986 (78.2)	52.3 (64.4)
Error model parameters		
a	2.22E-16	
b	39.2 (14.9)	
BIC	-1040.89	

*NC: Not computed

Supplementary Table S2. The final estimated parameters and bioavailability after oral administration of 2.5 µg, 5 µg and 10 µg of [^{13}C]-cyanocobalamin (n=9).

Participant ID*	Tk01	Tk02	F1	Tlag2	V	k	k12	k21	Bioavailability (%)
B12-DD-01_V1	7	4.04	0.628	4.81	7.99	0.0021	2.84	0.0967	29.2
B12-DD-01_V2	5.92	4.53	0.593	5	28.38	0.0021	6.24	0.0803	8.2
B12-DD-01_V3	4.41	2.81	0.597	5	8.52	0.002	3.87	0.0852	27.4
B12-DD-02_V1	5.42	4.64	0.661	4.47	3.01	0.0021	2.07	0.0917	101.1
B12-DD-02_V2	4.94	1.48	0.632	3.84	7.53	0.0021	3.82	0.0979	40.4
B12-DD-02_V3	5.59	2.65	0.591	4.34	3.78	0.0022	1.94	0.106	80.5
B12-DD-03_V1	6.54	2.17	0.568	5	16.62	0.0021	4.82	0.0855	11.9
B12-DD-03_V2	6.01	4.58	0.65	5.01	5.82	0.002	3.19	0.0814	33.9
B12-DD-03_V3	7.23	2.25	0.549	4.23	20.69	0.0021	5.22	0.0947	9.5
B12-DD-04_V1	6.14	6.51	0.608	4.44	48.63	0.0021	8.65	0.0794	4.9
B12-DD-04_V2	7.42	1.49	0.507	6	17.13	0.0021	4.48	0.0886	13.9
B12-DD-04_V3	4.8	8.58	0.656	4.52	21.19	0.0021	5.5	0.073	11.2
B12-DD-05_V1	7.13	2.68	0.528	5	20.64	0.0022	5.03	0.0895	10.9
B12-DD-05_V2	6	5.46	0.562	5	31.55	0.0017	6.65	0.0693	7.2
B12-DD-05_V3	6.85	2.92	0.595	4.22	10.19	0.0021	3.42	0.0971	22.2
B12-DD-06_V1	6	8.54	0.679	5	17.38	0.0027	5.39	0.0705	11.1
B12-DD-06_V2	6.95	2	0.567	5	12.8	0.0021	3.74	0.0943	15.0
B12-DD-06_V3	6.68	1.02	0.585	5	6.58	0.0021	2.83	0.0918	29.3
B12-DD-07_V1	6.99	3.43	0.578	4.42	24.14	0.0021	5.42	0.0962	10.1
B12-DD-07_V2	4.87	3.26	0.632	4.52	3.71	0.0021	2.07	0.0985	65.5
B12-DD-07_V3	7	3.68	0.626	4.81	6.38	0.0021	2.52	0.098	38.1
B12-DD-08_V1	4.63	2.93	0.632	4.82	2.81	0.0021	1.9	0.1	96.0
B12-DD-08_V2	3.91	3.06	0.578	5	7.15	0.0018	4.46	0.0549	37.7
B12-DD-08_V3	6.68	1.74	0.541	4.25	9.46	0.0021	3.16	0.1	28.5
B12-DD-09_V1	7.39	3.52	0.563	4.4	38.01	0.0021	7.31	0.0949	6.9
B12-DD-09_V2	5.5	6.83	0.681	4.39	3.91	0.0021	2.42	0.0796	67.4
B12-DD-09_V3	7.22	2.77	0.517	4.23	13.55	0.0022	3.97	0.0991	19.4

*Randomization sequence for oral dose of [^{13}C]-cyanocobalamin at each visit is given in supplementary figure S1.
